# Supplementary material for: Processive dynamics of the usher assembly platform during uropathogenic Escherichia coli P pilus biogenesis
Source: Nat Commun. 2021 Sep 1;12:5207. doi: 10.1038/s41467-021-25522-6 (PMC8410936; doi:10.1038/s41467-021-25522-6)
Supplement: Supplementary file 1 — Supplementary Information [file 41467_2021_25522_MOESM1_ESM.pdf]

SUPPLEMENTARY INFORMATION

Processive Dynamics of the Usher Assembly Platform During Uropathogenic *Escherichia coli* P Pilus Biogenesis

Minge Du, Zuanning Yuan, Glenn T. Werneburg, Nadine S. Henderson, Hemil Chauhan, Amanda Kovach, Gongpu Zhao, Jessica Johl, Huilin Li, David G. Thanassi

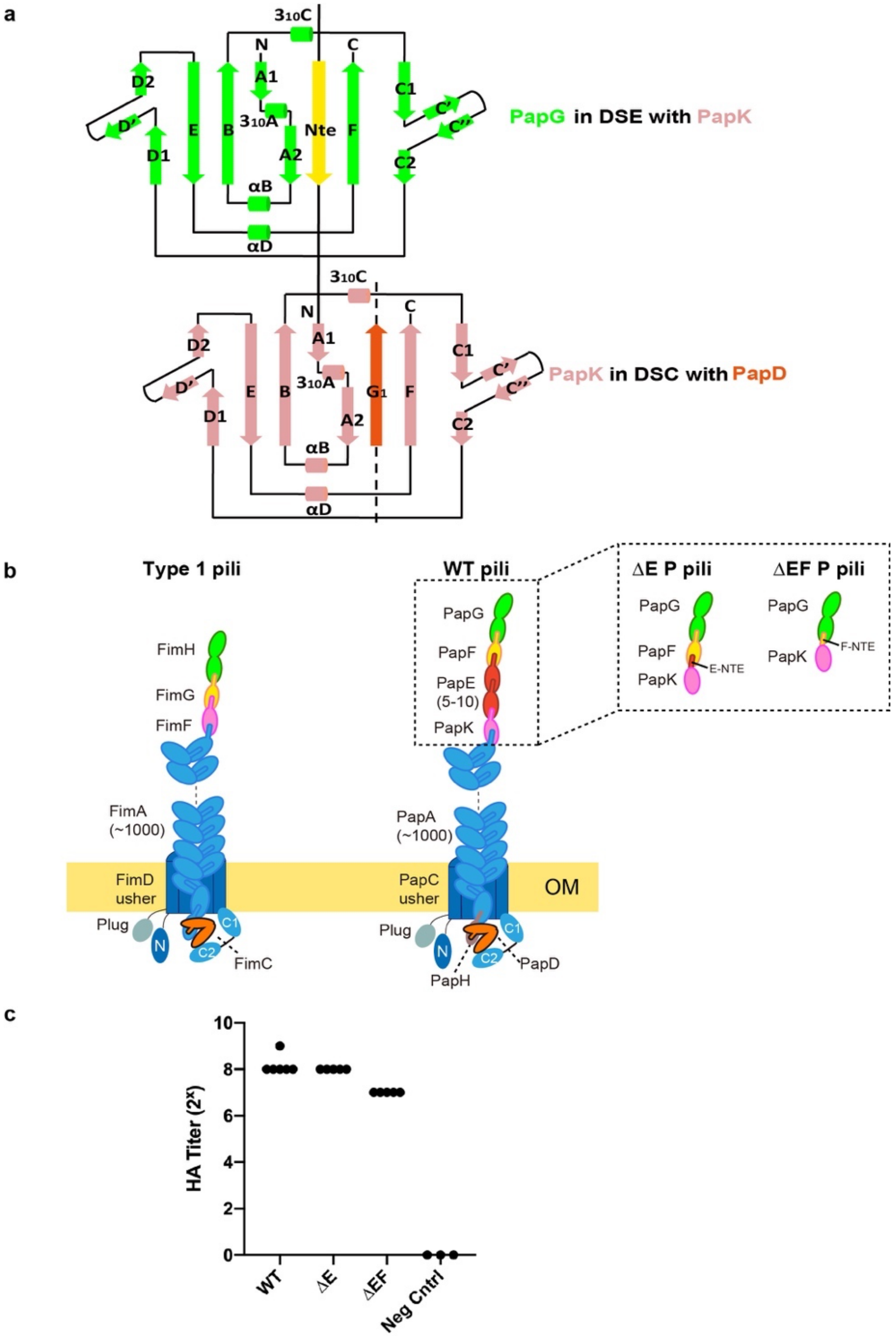

Supplementary Figure 1. Schematics showing donor strand complementation and exchange interactions, type 1 and P pili, and the engineered ∆E and ∆EF P pilus variants. a, Donor strand complementation (DSC): In the periplasm, the PapD chaperone donates its G1 ß-strand to complete the immunoglobulin (Ig)-like fold of newly translocated pilus subunits (PapK in this case). The PapD ß-strand is inserted parallel to the subunit F strand, in a non-canonical manner. Donor strand exchange (DSE): the chaperone donor strand of the preceding subunit (PapG in this case) is replaced by the N-terminal extension (NTE) of the incoming

subunit (PapK with engineered PapF NTE). The NTE is inserted anti-parallel to the subunit F strand, forming a canonical Ig fold. **b,** Cartoons showing the wild-type type I and P pilus assemblies, and the ΔE and ΔEF engineered P pilus variants. OM, outer membrane. **c,** Hemagglutination assay results comparing bacteria expressing wild-type (WT), ΔE, ΔEF or no (Neg Cntrl) P pili. HA titers represent the greatest fold dilution of bacteria able to agglutinate human red blood cells and were calculated from at least three independent experiments, with three replicates per experiment.

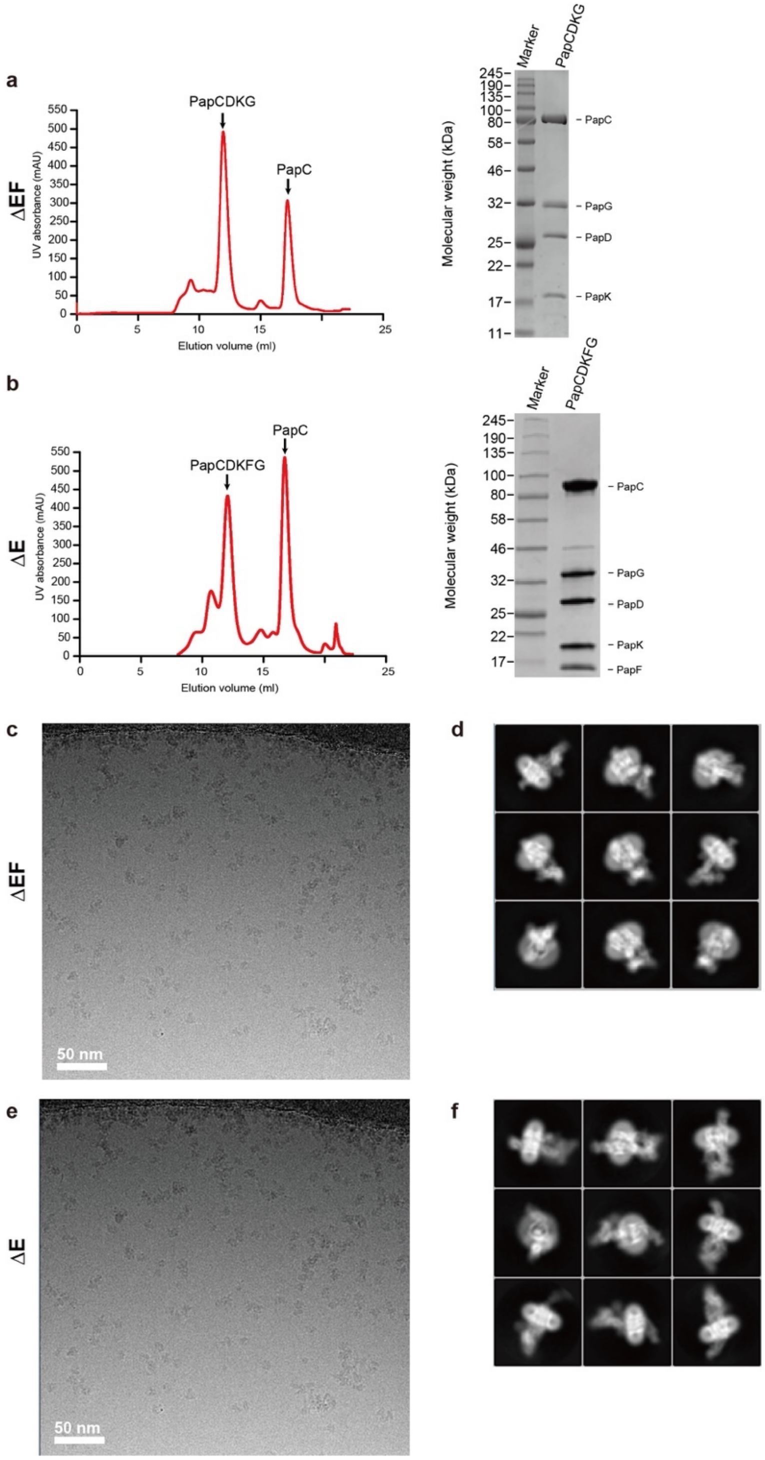

Supplementary Figure 2. Cryo-EM of P pilus assembly intermediates. a, Gel filtration profile and SDS-PAGE of ΔEF P pilus complexes. **b,** Gel filtration profile and SDS-PAGE of ΔE P pilus complexes. **c,** Raw image of the purified ΔEF P pilus complexes and **d,** selected 2D class averages showing the presence of many different views. **e,** Raw image of the purified ΔE P pilus complexes and **f,** selected 2D class averages showing that different views of the complex are well sampled.

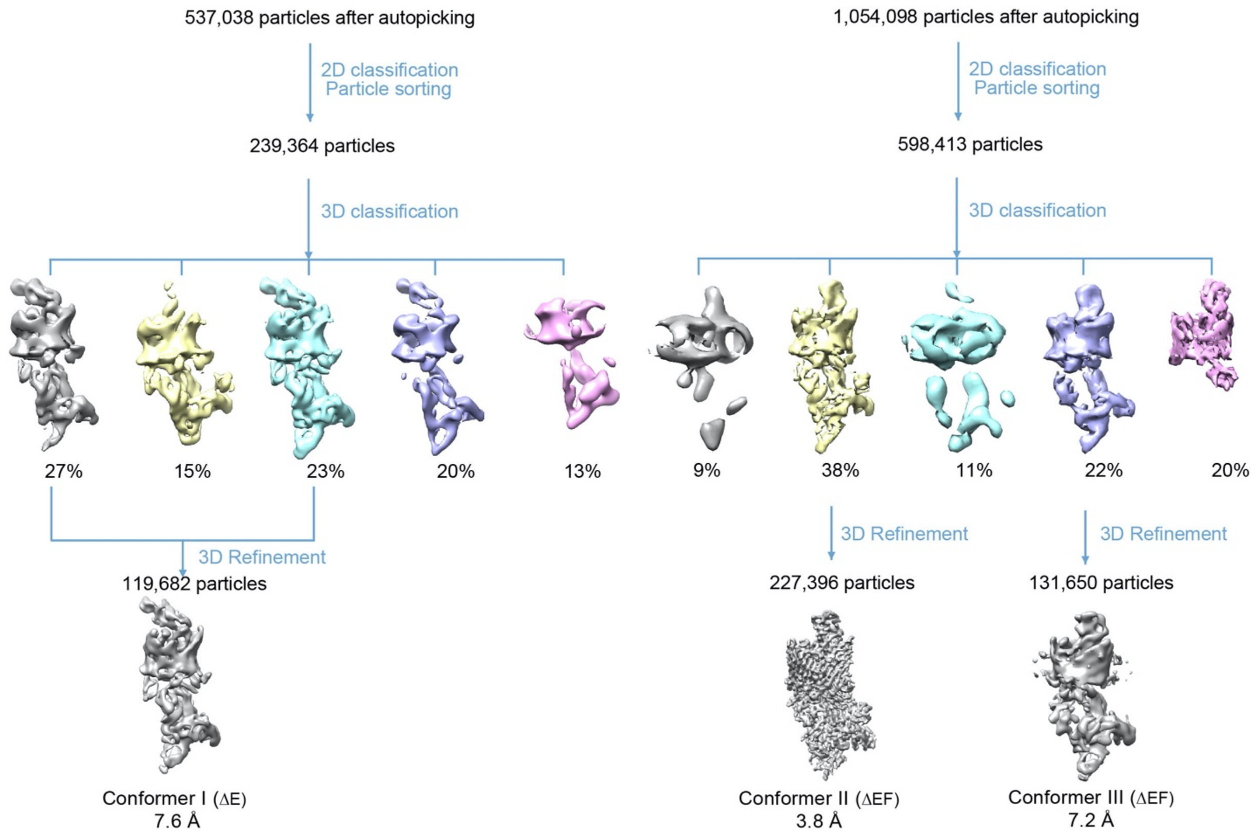

Supplementary Figure 3. 3D classification scheme. For ΔE, over a half million raw particles were selected from drift corrected electron micrographs. 2D and 3D classification resulted in three 3D maps that were of the expected shape and the structure appeared complete, and two other maps that were either partial structures or distorted. The three good maps ΔE were combined. Refinement with 119,682 combined particles led to the 7.6 Å Conformer I 3D map. **b,** For ΔEF, similar processes led to two final EM maps: a 3.8 Å map for Conformer II and a 7.2 Å map for Conformer III.

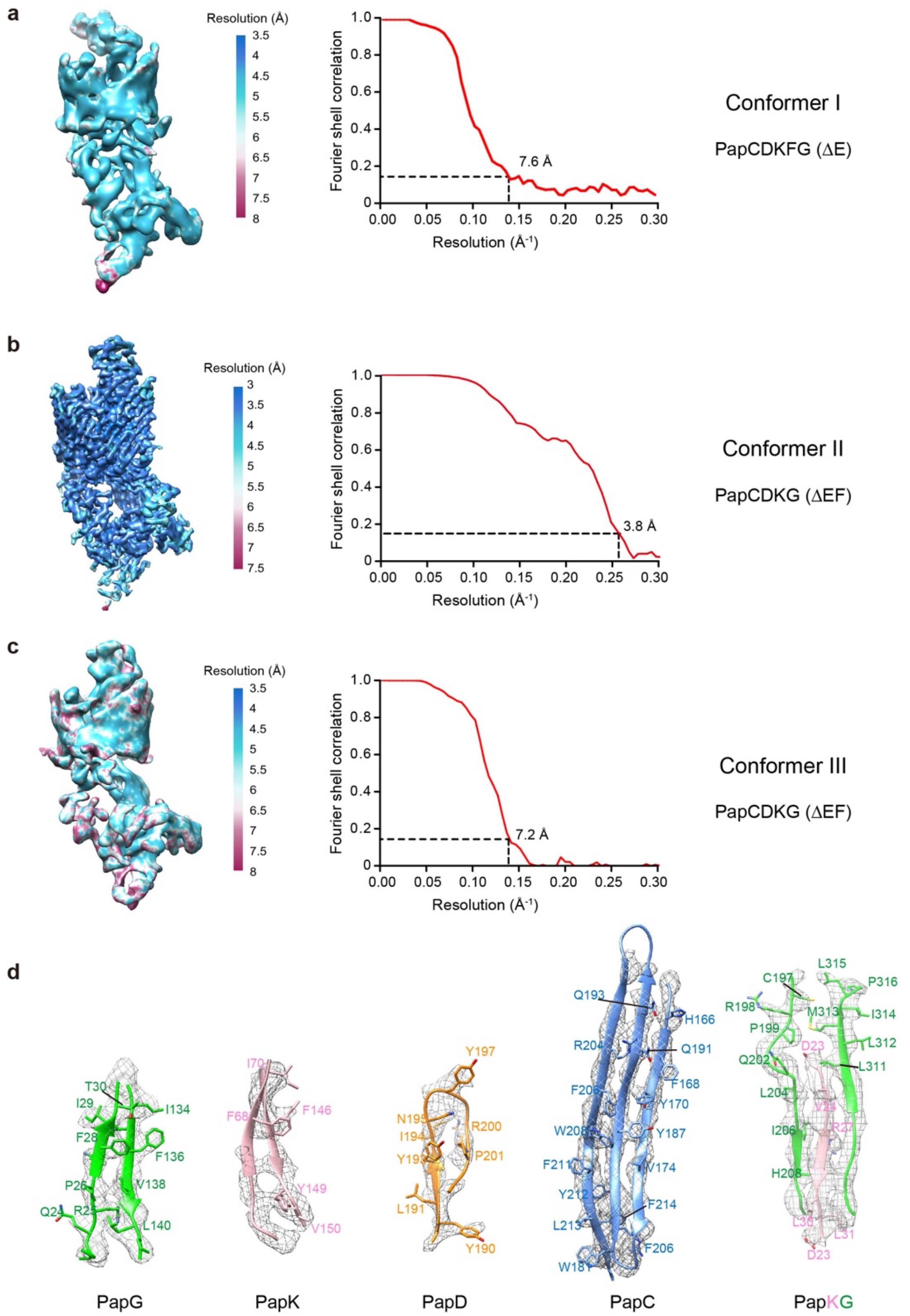

Supplementary Figure 4. Resolution estimation of the 3D EM maps. a-c, Gold standard Fourier shell correlation estimation at the 0.143 correlation threshold and the local resolution estimation of the P pilus tip complexes in Conformers I, II and III. **d,** Selected sheet regions of PapG, PapK, PapD, PapC and PapKG showing the densities of large side chains.

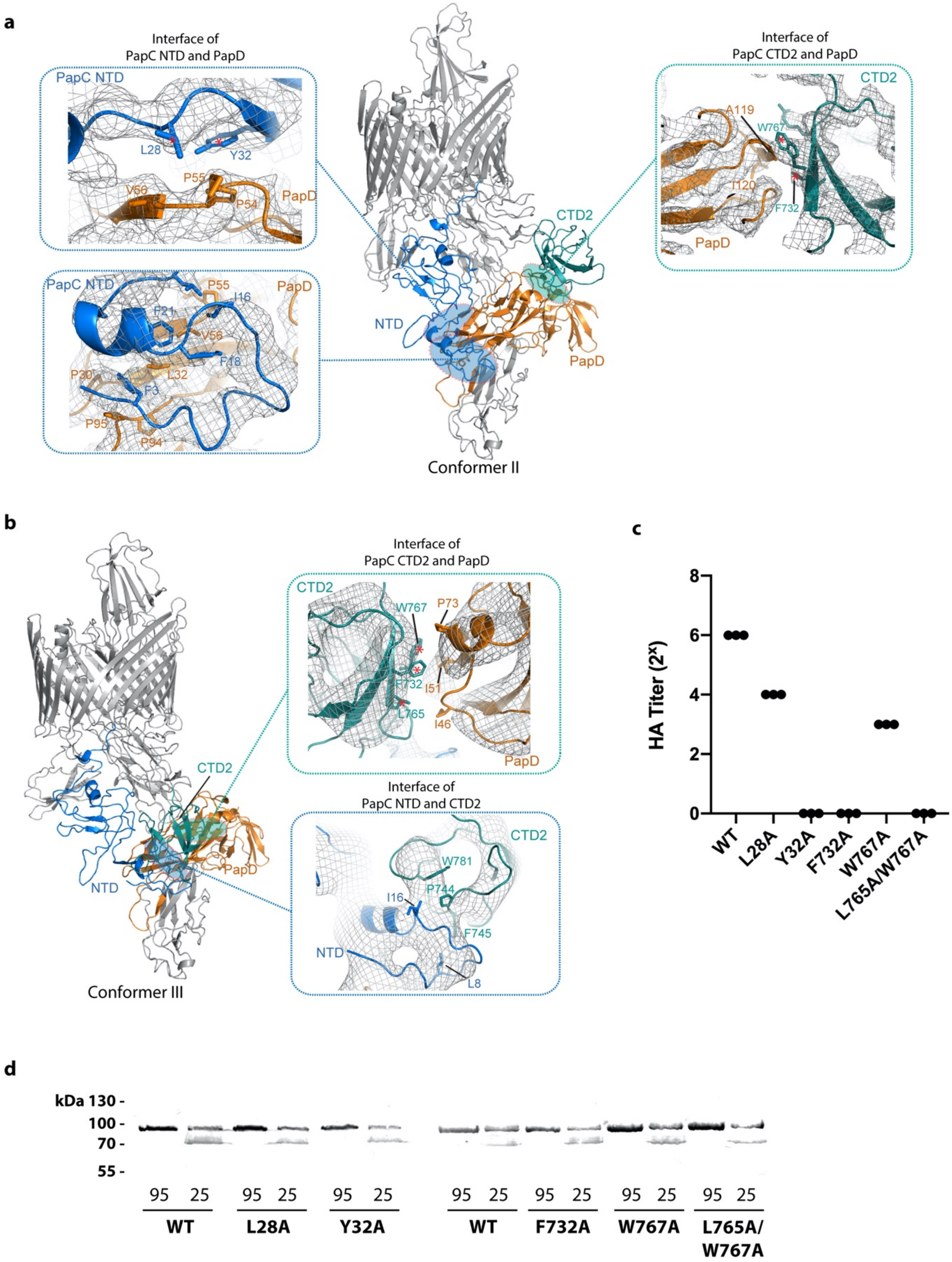
a
Interface of
PapC NTD and PapD
PapC NTD
L28
Y32
P55
V56
P54
PapD
PapC NTD
P55
I16
PapD
F21
V56
F18
P30
L32
F3
P95
P94
NTD
CTD2
PapD
Conformer II
Interface of
PapC CTD2 and PapD
CTD2
A119
W767
I120
F732
PapD
b
Conformer III
CTD2
PapD
NTD
Interface of
PapC CTD2 and PapD
CTD2
W767
P73
F732
I51
L765
I46
PapD
Interface of
PapC NTD and CTD2
CTD2
W781
P744
I16
F745
NTD
L8
c
HA Titer (2ˣ)
8
6
4
2
0
WT
L28A
Y32A
F732A
W767A
L765A/W767A
d
kDa 130 -
100 -
70 -
55 -
95 25 95 25 95 25 95 25 95 25 95 25 95 25
WT
L28A
Y32A
WT
F732A
W767A
L765A/
W767A

Supplementary Figure 5. PapC NTD and CTD2 and PapD interactions in ∆EF Conformers II and III. a, The interfaces in Conformer II between PapC NTD and PapD, and between PapC CTD2 and PapD, are shown in cartoon view. **b**, The interfaces in Conformer III between PapC CTD2 and PapD, and between PapC NTD and CTD2, are shown in cartoon view. **c,** Hemagglutination assay results comparing the ability of wild-type (WT) PapC and PapC point mutants (denoted by red asterisks in panels **a** and **b**) to assemble adhesive P pili. HA titers represent the greatest fold dilution of bacteria able to agglutinate human red blood cells and were calculated from three independent experiments, with three replicates per experiment. **d**, Expression levels and folding of the PapC mutants analyzed in panel **c**. OM fractions from bacteria expressing WT or mutated PapC were incubated at 25°C or 95°C in SDS-PAGE sample buffer, subjected to SDS-PAGE, and blotted with anti-His-tag antibodies. The presence of the faster-migrating folded monomer band in the 25°C-treated samples indicates proper folding of the usher in the OM. The corresponding full blot scans are shown in Supplementary Fig. 10.

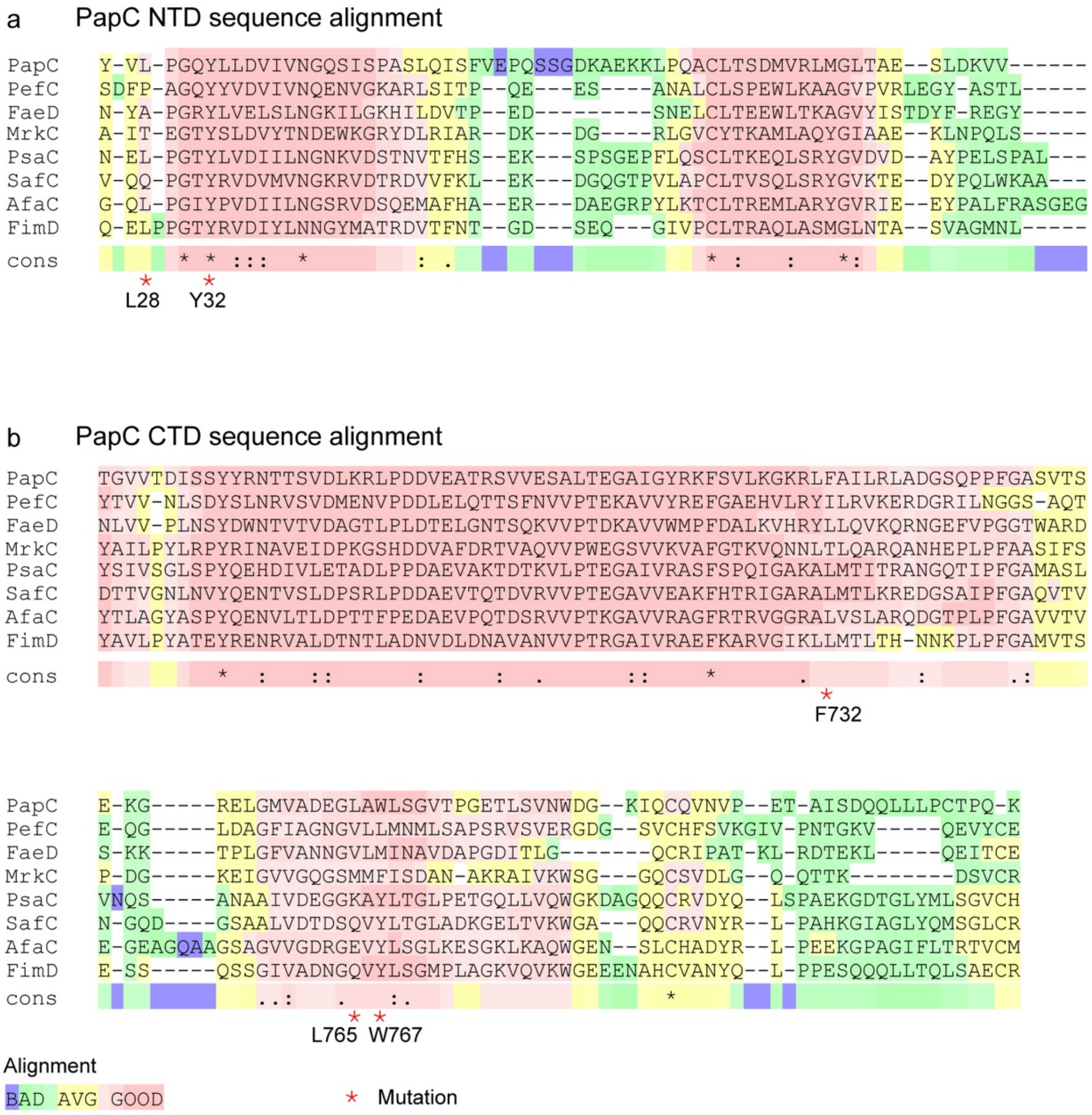

Supplementary Figure 6. Sequence conservation of the interacting interfaces at the PapC usher N- and C-terminal domains. Sequence alignment of the (**a**) NTD and (**b**) CTD of ushers PapC, FimD, MrkC and AfaC from *E. coli*, SafC and PefC from *Salmonella enterica*, FaeD from *Erwinia amylovora*, and PsaC from *Yersinia pestis*. Residues involved in interactions between the PapC NTD and PapD, and PapC CTD2 and PapD, as tested experimentally in this study, are labeled with red stars.

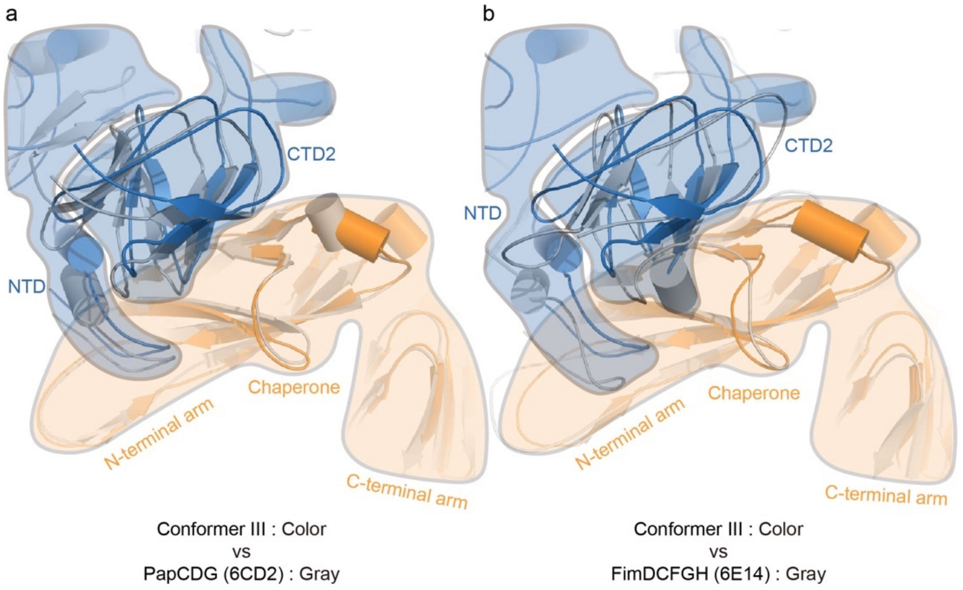

Supplementary Figure 7. Comparison of the PapC NTD-PapD chaperone-PapC CTD2 interface in Conformer III, PapCDG and FimDCFGH. a,b, the NTD-PapD-CTD2 interface in Conformer III is similar to the interfaces observed in the PapCDG crystal structure and the type 1 pilus FimD-tip complex.

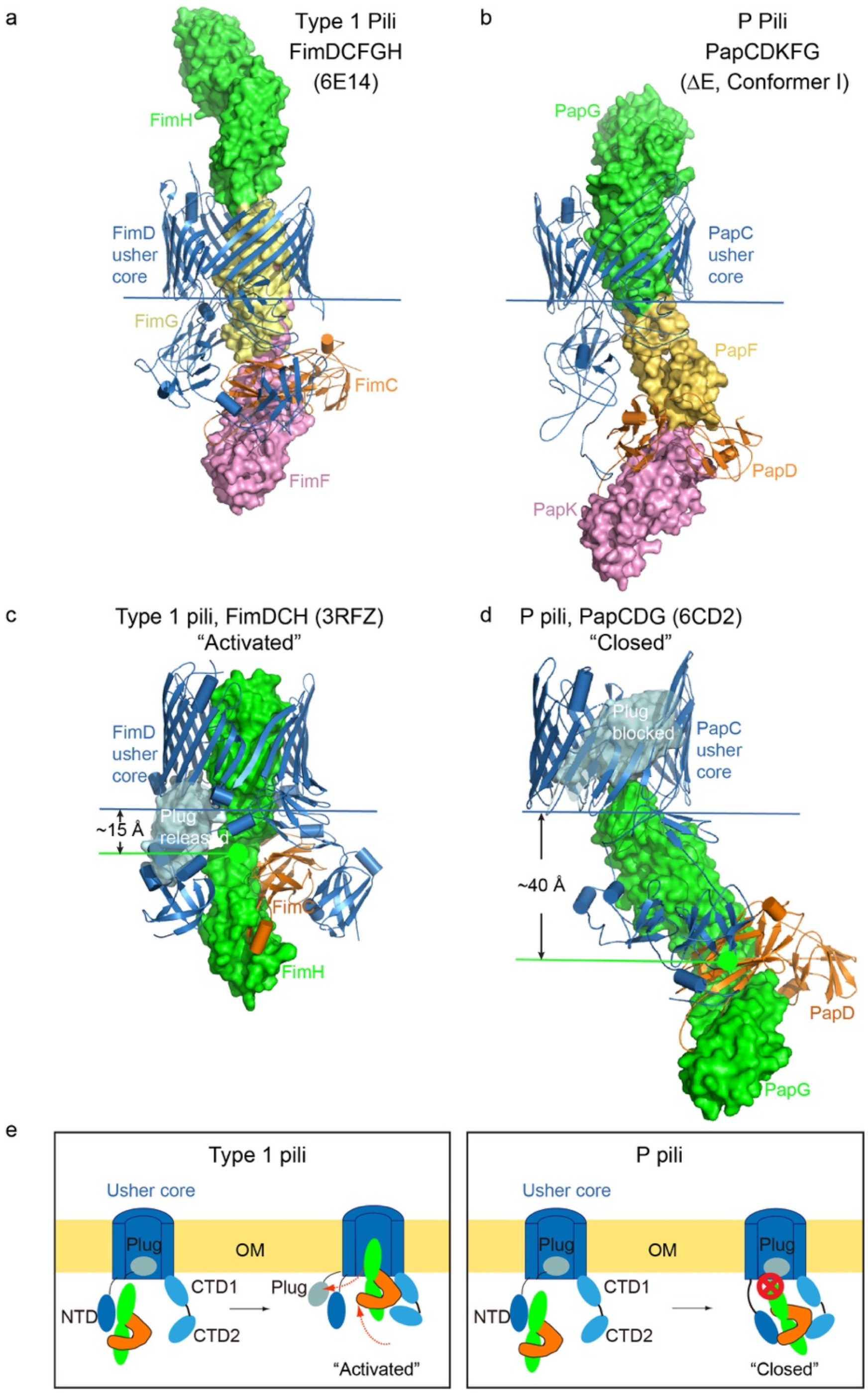

Supplementary Figure 8. The PapC and FimD ushers adopt different activation mechanisms. a,b, Comparison of the three-subunit FimDCFGH and PapCDKFG type 1 and P pilus assembly intermediates shows that the distance between the last incorporated subunit and the periplasmic face of the usher ß-barrel channel differs significantly. **c-d,** In the one-subunit FimDCH and PapCDG type 1 and P pilus complexes, binding of the FimH adhesin activates the FimD usher, whereas binding of the PapG adhesin does not activate the PapC usher. **e,** Models comparing chaperone-adhesin binding to the FimD and PapC ushers. OM, outer membrane.

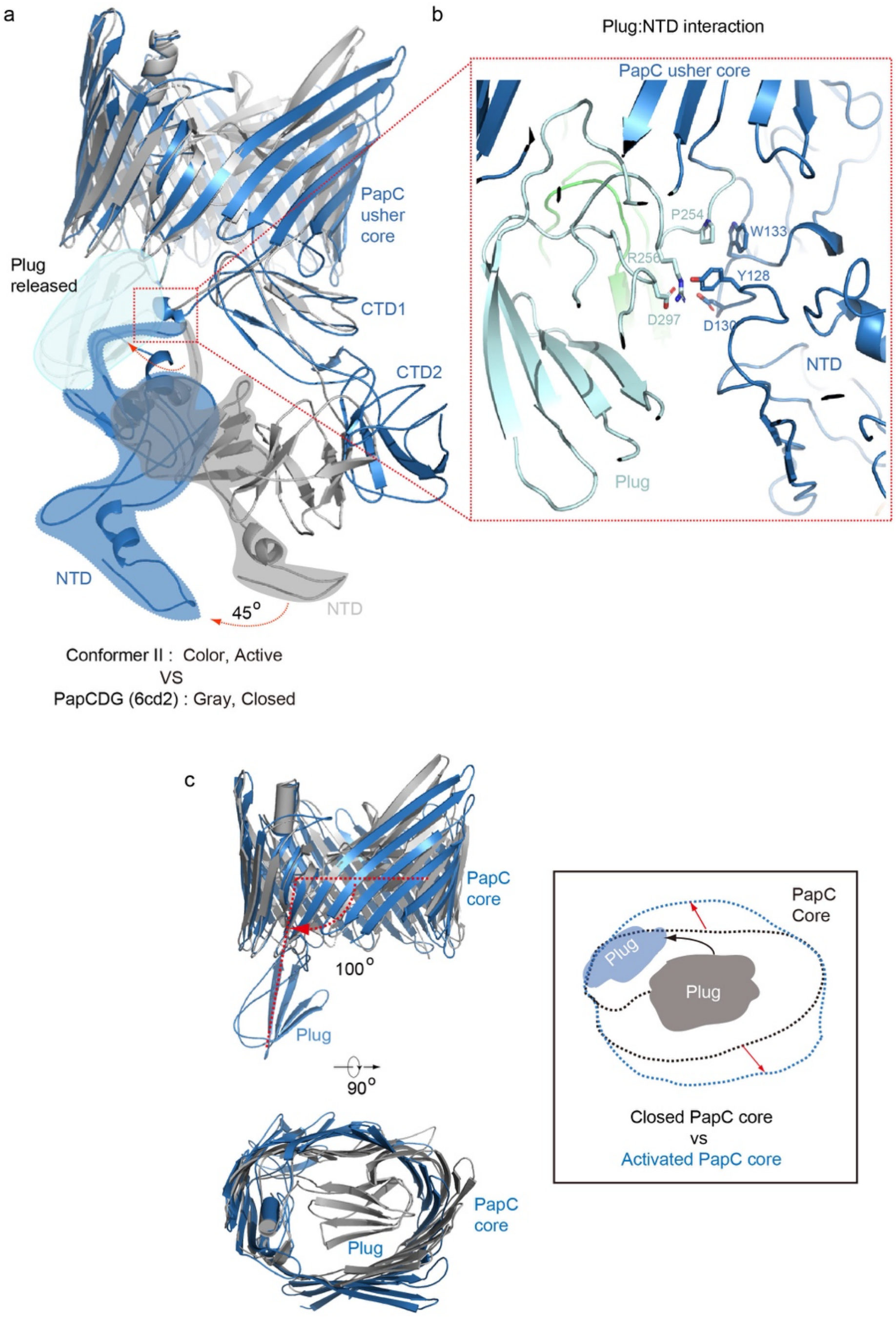

Supplementary Figure 9. Comparison between ∆EF Conformer II and PapCDG (6CD2). a, Overlap of PapC in Conformer II and the PapCDG crystal structure in color and gray cartoons, respectively. The NTD rotates by ~45°. **b,** Plug-NTD interaction in Conformer II and the residues included in the interaction. **c,** Conformational change between the closed PapC core in PapCDG and the activated PapC core in Conformer II. The plug domain rotates by ~100°.

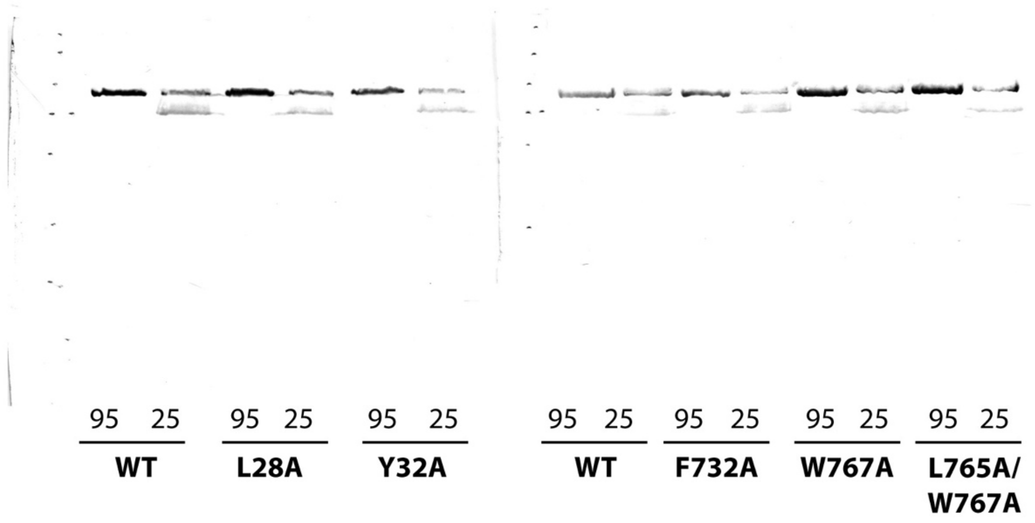

Supplementary Figure 10. Full blot scans corresponding to Supplementary Fig. 5d.

Supplementary Table 1. Cryo-EM data collection and refinement statistics

**Data Collection**	**∆E, PapCDK$_{E\text{-}NTE}$FG**	**∆EF, PapCDK$_{F\text{-}NTE}$G**	
	Conformer I	Conformer II	Conformer III
EM equipment	FEI Titan Krios	FEI Titan Krios	
Voltage (kV)	300	300	
Detector	Gatan K2	Gatan K2	
Pixel size (Å)	1.029	1.029	
Electron dose (e^-/Å^2)	60	60	
Defocus range (–µm)	1.5-2.5	1.5-2.5	
**Reconstruction**			
Software	RELION 3.0	RELION 3.0	
Number of used particles	119,682	227,396	131,650
Resolution (Å)	7.6	3.8	7.2
Map sharpening B-factor (Å^2)	-229	-115	-276
**Model composition**			
Peptide chains	5	4	4
Protein residues	1525	1493	1493
**R.m.s deviations**			
Bonds length (Å)	0.009	0.008	0.008
Bonds Angle (°)	1.38	1.74	1.68
**Ramachandran plot**			
Preferred (%)	89.91	94.61	94.20
Allowed (%)	9.29	5.25	5.73
Outlier (%)	0.80	0.13	0.07
**Validation**			
Molprobity score	2.06 (72%)	1.77 (87%)	1.79 (86%)
Rotamer outliers (%)	0.35	0.00	0.00

Supplementary Table 2. Strains and plasmids used in this study

Strain or construct[a]	Relevant characteristic(s)	Reference or source
<u>Strains</u>		
DH5α	*hsdR, recA, endA*	[1]
Tuner	OmpT⁻ Lon⁻	Novagen
SF100	Δ*ompT*	[2]
AAEC185	Δ*fim*	[3]
<u>Plasmids</u>		
pMJ2	*papC⁻ pap* operon, P_{trc}, Tet^r	[4]
pKD101	PapC, P_{tac}, Kan^r	[5]
pTN46	PapA in vector pACYC184, P_{ara}, Clm^r	T. Ng and D.G.T., unpublished
pJL01	*papDJKEFG* with His-tagged PapD, P_{trc}, Amp^r, derived from plasmid pFJ6[6]	J. LeBarron and D.G.T., unpublished
pGW990	Δ*papE*, PapK NTE replaced with PapE NTE, in pJL01	This study
pGW992	Δ*papEF*, PapK NTE replaced with PapF NTE, in pJL01	This study
pDG2	His-tagged PapC, P_{ara}, Amp^r	[7]
pNH577	PapC L28A in pDG2	This study
pNH580	PapC Y32A in pDG2	This study
pNH583	PapC F732A in pDG2	This study
pNH584	PapC W767A in pDG2	This study
pNH585	PapC L765A/W767A in pDG2	This study

Amp^r, ampicillin resistance; Clm^r, chloramphenicol resistance; Kan^r, kanamycin resistance; Tet^r, tetracycline resistance; P_{ara}, arabinose-inducible promoter, P_{trc}, IPTG-inducible promoter.

Supplementary Table 3. Primers used in this study

Plasmid	Mutation	Method	Primer
pGW990, pGW992	Δ*papE*	SLIM	5'TGTACAAAAATTTTGCTAACGATATGTCAATTCAAACGTGGCTGTTGC
			5'ATATGTCAATTCAAACGTGGCTGTTGC
			5'CGTTAGCAAAATTTTTGTACAAAATGTTTTCTGTACCGCTCTCCGGAG
			5'AAATGTTTTCTGTACCGCTCTCCGGAG
pGW992	Δ*pap*F	SLIM	5'TAAACGAATCATTCCCCCTCCGGAGAGCGGTACAGAAAACA
			5'GGAGAGCGGTACAGAAAACA
			5'GGAGGGGGAATGATTCGTTTACACGGCCAGTATGAGCATGA
pGW990	PapK NTE replaced with PapE NTE	SLIM	5'ACAGGCAGGAATAATCAGTTTTCCTCTGAAGGTCAGATTATCAACGGCTATTGCCTGTCCGGCAGA
			5'GGCTATTGCCTGTCCGGCAGA
			5'GTTGATAATCTGACCTTCAGAGGAAAACTGATTATTCCTGCCTGTCATGTGTCCGGTGACAGTCTG
			5'CATGTGTCCGGTGACAGTCTG
pGW992	PapK NTE replaced with PapF NTE	SLIM	5'GCATGGGGGGATATAAACATTCCCCCTGATGTTAATCTGCACATCGGCTATTGCCTGTCCGGCAGA
			5'GGCTATTGCCTGTCCGGCAGA
			5'GATGTGCAGATTAACATCAGGGGGAATGTTTATATCCCCCCATGCCATGTGTCCGGTGACAGTCTG
			5'CATGTGTCCGGTGACAGTCTG
pNH577	PapC L28A	QC	5'GAAGCCGGCTATGTTGCGCCGGGGCAATATCTTCTG
pNH580	PapC Y32A	QC	5'GGCTATGTTCTGCCGGGGCAAGCTCTTCTGGATGTGATTG
pNH583	PapC F732A	QC	5'GTGCTTAAAGGGAAACGTCTGGCTGCAATACTGCGTCTTGCTGATG
pNH585	PapC L765A	QC	5'GCCGACGAAGGCGCTGCCGCGCTGAGTGG
pNH584	PapC W767A	QC	5'CGAAGGCCTTGCCGCGCTGAGTGGCGTGAC

Site-directed, Ligase-independent Mutagenesis (SLIM) was performed as described[8,9]. QuikChange (QC) reactions were performed using the QuikChange XL protocol (Stratagene). Each QC reverse-primer is the complement of the listed primer sequence.

Supplementary References

1. Grant S. G., Jessee J., Bloom F. R. & Hanahan D. Differential plasmid rescue from transgenic mouse DNAs into *Escherichia coli* methylation-restriction mutants. *Proc. Natl. Acad. Sci. USA* **87**, 4645-4649 (1990).

2. Baneyx F. & Georgiou G. In vivo degradation of secreted fusion proteins by the *Escherichia coli* outer membrane protease OmpT. *J. Bacteriol.* **172**, 491-494 (1990).

3. Blomfield I. C., McClain M. S. & Eisenstein B. I. Type 1 fimbriae mutants of *Escherichia coli* K12: characterization of recognized afimbriate strains and construction of new fim deletion mutants. *Mol. Microbiol.* **5**, 1439-1445 (1991).

4. Thanassi D. G., Saulino E. T., Lombardo M. J., Roth R., Heuser J. & Hultgren S. J. The PapC usher forms an oligomeric channel: implications for pilus biogenesis across the outer membrane. *Proc. Natl. Acad. Sci. USA* **95**, 3146-3151 (1998).

5. Dodson K. W., Jacob-Dubuisson F., Striker R. T. & Hultgren S. J. Outer-membrane PapC molecular usher discriminately recognizes periplasmic chaperone-pilus subunit complexes. *Proc. Natl. Acad. Sci. USA* **90**, 3670-3674 (1993).

6. Jacob-Dubuisson F., Heuser J., Dodson K., Normark S. & Hultgren S. Initiation of assembly and association of the structural elements of a bacterial pilus depend on two specialized tip proteins. *EMBO J.* **12**, 837-847 (1993).

7. Li H. et al. The outer membrane usher forms a twin-pore secretion complex. *J. Mol. Biol.* **344**, 1397-1407 (2004).

8. Chiu J., March P. E., Lee R. & Tillett D. Site-directed, ligase-independent mutagenesis (SLIM): a single-tube methodology approaching 100% efficiency in 4 h. *Nucleic Acids Res.* **32**, e174 (2004).

9. Chiu J., Tillett D., Dawes I. W. & March P. E. Site-directed, ligase-independent mutagenesis (SLIM) for highly efficient mutagenesis of plasmids greater than 8kb. *J. Microbiol. Methods* **73**, 195-198 (2008).
