# Supplementary material for: Processive dynamics of the usher assembly platform during uropathogenic Escherichia coli P pilus biogenesis
Source: Nat Commun. 2021 Sep 1;12:5207. doi: 10.1038/s41467-021-25522-6 (PMC8410936; doi:10.1038/s41467-021-25522-6)
Supplement: Supplementary file 3 — Description of Additional Supplementary Files [file 41467_2021_25522_MOESM3_ESM.pdf]

Description of Additional Supplementary Files

File Name: Supplementary Movie 1
Description: Morph from ΔEF (PapCDK) Conformer II to Conformer III. The movie is in the side view and starts with Conformer II and morphs into Conformer III. PapC CTD2 shifts to the N-terminal arm of the first lobe of the PapD chaperone, whereas PapC NTD2 and the PapDK complex undergo converse lateral movements. The movie was prepared using UCSF Chimera.

File Name: Supplementary Movie 2
Description: Morph from ΔEF (PapCDKG) Conformer III to ΔE (PapCDKFG) Conformer I, with an emphasis on how the PapG adhesin passes through the PapC usher secretion channel. For clarity, the tip subunits PapF and PapK, as well as the usher NTD and CTDs, are removed. The movie starts with Conformer III and morphs into Conformer I in the side view. PapG moves outward and through the PapC channel with the PapG lectin domain bending back by 60º toward the ß-barrel channel. Next, the scene rotates 90° to the top view, showing that PapG rolls counterclockwise while emerging from the PapC channel. The movie was prepared using UCSF Chimera.
