# Supplementary material for: Processive dynamics of the usher assembly platform during uropathogenic Escherichia coli P pilus biogenesis
Source: Nat Commun. 2021 Sep 1;12:5207. doi: 10.1038/s41467-021-25522-6 (PMC8410936; doi:10.1038/s41467-021-25522-6)
Supplement: Supplementary file 6 — Reporting Summary [file 41467_2021_25522_MOESM6_ESM.pdf]

nature portfolio

Corresponding author(s): Hulin Li and David G. Thanassi

Last updated by author(s): Jul 27, 2021

Reporting Summary

Nature Portfolio wishes to improve the reproducibility of the work that we publish. This form provides structure for consistency and transparency in reporting. For further information on Nature Portfolio policies, see our Editorial Policies and the Editorial Policy Checklist.

Statistics

For all statistical analyses, confirm that the following items are present in the figure legend, table legend, main text, or Methods section.

n/a	Confirmed	
☐	☒	The exact sample size (*n*) for each experimental group/condition, given as a discrete number and unit of measurement
☐	☒	A statement on whether measurements were taken from distinct samples or whether the same sample was measured repeatedly
☒	☐	The statistical test(s) used AND whether they are one- or two-sided<br>*Only common tests should be described solely by name; describe more complex techniques in the Methods section.*
☒	☐	A description of all covariates tested
☒	☐	A description of any assumptions or corrections, such as tests of normality and adjustment for multiple comparisons
☒	☐	A full description of the statistical parameters including central tendency (e.g. means) or other basic estimates (e.g. regression coefficient) AND variation (e.g. standard deviation) or associated estimates of uncertainty (e.g. confidence intervals)
☒	☐	For null hypothesis testing, the test statistic (e.g. *F*, *t*, *r*) with confidence intervals, effect sizes, degrees of freedom and *P* value noted<br>*Give P values as exact values whenever suitable.*
☒	☐	For Bayesian analysis, information on the choice of priors and Markov chain Monte Carlo settings
☒	☐	For hierarchical and complex designs, identification of the appropriate level for tests and full reporting of outcomes
☒	☐	Estimates of effect sizes (e.g. Cohen's *d*, Pearson's *r*), indicating how they were calculated

Our web collection on statistics for biologists contains articles on many of the points above.

Software and code

Policy information about availability of computer code

Data collection	Cryo-EM data collection used SerialEM (version 3.8.8) in Titan Krios and Arctica.
Data analysis	For analysis and modeling of cryo-EM data, the following software was used: Motioncorr 2.0, CTFFIND 4.1, Relion-3.0, Swiss model online server, COOT (version 0.8.9.1), ChimeraX 1.0, PyMOL 2.2.0, PHENIX (version 1.14-3260) and MolProbity (version 4.5).

For manuscripts utilizing custom algorithms or software that are central to the research but not yet described in published literature, software must be made available to editors and reviewers. We strongly encourage code deposition in a community repository (e.g. GitHub). See the Nature Portfolio guidelines for submitting code & software for further information.

Data

Policy information about availability of data

All manuscripts must include a data availability statement. This statement should provide the following information, where applicable:

- Accession codes, unique identifiers, or web links for publicly available datasets
- A description of any restrictions on data availability
- For clinical datasets or third party data, please ensure that the statement adheres to our policy

The cryo-EM 3D maps of the PapC-tip complex generated in this study have been deposited at the EMDB database with accession codes EMD-23341 [https://www.ebi.ac.uk/emdb/entry/EMD-23341] (Conformer I), EMD-23339 [https://www.ebi.ac.uk/emdb/entry/EMD-23339] (Conformer II) and EMD-23340 [https://www.ebi.ac.uk/emdb/entry/EMD-23340] (Conformer III). The corresponding atomic models were deposited at the RCSB PDB with accession codes 7LHI [https://www.rcsb.org/structure/7LHI] (Conformer I), 7LHG [https://www.rcsb.org/structure/7LHG] (Conformer II), and 7LHH [https://www.rcsb.org/structure/7LHH] (Conformer III).

RCSB PDB accession codes for previously reported structures cited in this study are as follows: PapCDG (6CD2) [https://www.rcsb.org/structure/6CD2], PapDK (1PDK) [https://www.rcsb.org/structure/1PDK], PapF (2W07) [https://www.rcsb.org/structure/2W07], FimDCFGH (6E14) [https://www.rcsb.org/structure/6E14], FimDCH (3RFZ) [https://www.rcsb.org/structure/3RFZ].

Field-specific reporting

Please select the one below that is the best fit for your research. If you are not sure, read the appropriate sections before making your selection.

☒ Life sciences ☐ Behavioural & social sciences ☐ Ecological, evolutionary & environmental sciences

For a reference copy of the document with all sections, see nature.com/documents/nr-reporting-summary-flat.pdf

Life sciences study design

All studies must disclose on these points even when the disclosure is negative.

Sample size	We collected 14,722 raw movie micrographs of the ΔEF complexes and 25,183 raw movie micrographs of the ΔE complex. The sample size was deemed sufficient as it resulted in 3D maps at the anticipated resolution.
Data exclusions	For the ΔEF complex, the template for automatic picking was generated from a 2D average of about ~10,000 manually picked particles in different views. Automatic particle selection was performed for the entire data set, and 1,054,098 particles were initially picked. We then carefully inspected the selected particles, removed "bad" ones and re-picked some initially missed "good" ones, and sorted the remaining good particles by similarity to the 2D references, in which the bottom 10% of particles with the lowest z-scores were removed from the particle pool. 2D classification of all good particles was performed and particles in the classes with unrecognizable features by visual inspection were removed. A total of 598,413 particles were used for further 3D classification. We derived five 3D models from the dataset and chose the two best models for the final refinement. The other three models were distorted and those particles were discarded.<br><br>For the ΔE complex, we used a similar process as for the ΔEF complex. After 2D classification, 239,364 particles were used for further 3D classification. We derived five 3D models from the dataset and combined three similar models for the final refinement. The other two models were distorted and those particles were discarded.
Replication	For hemagglutination assays, HA titers were calculated from at least three independent experiments, with three replicates per experiment. All attempts at replication were successful for all experiments reported in the study.
Randomization	Randomization is not relevant to our structural biology study.
Blinding	Blinding is not relevant to our structural biology study.

Reporting for specific materials, systems and methods

We require information from authors about some types of materials, experimental systems and methods used in many studies. Here, indicate whether each material, system or method listed is relevant to your study. If you are not sure if a list item applies to your research, read the appropriate section before selecting a response.

Materials & experimental systems

n/a	Involved in the study	
☐	☒	Antibodies
☒	☐	Eukaryotic cell lines
☒	☐	Palaeontology and archaeology
☒	☐	Animals and other organisms
☐	☒	Human research participants
☒	☐	Clinical data
☒	☐	Dual use research of concern

Methods

n/a	Involved in the study	
☒	☐	ChIP-seq
☒	☐	Flow cytometry
☒	☐	MRI-based neuroimaging

Antibodies

Antibodies used	The anti-His Ab we used is from Biolegend (San Diego, CA). The name of the antibody is "purified anti-His epitope tag", clone 6-His, catalog number 906102, lot number B233197. We used the antibody at 1:1,000 dilution.
Validation	This is a widely used, commercially produced and validated antibody. The manufacturer provides validation information on their website (https://www.biolegend.com/en-us/products/purified-anti-6-his-epitope-tag-antibody-11247) and states that "Each lot of this antibody is quality control tested by Western blotting." The blot shown in Fig. S5d of our manuscript shows proper reaction of the antibody with only the His-tagged protein of the expected size and behavior.

Human research participants

Policy information about studies involving human research participants

Population characteristics	Human red blood cells were obtained from donors with informed consent according to a protocol approved by the Institutional Review Board of Stony Brook University. Blood donors were recruited by word of mouth and reflected the population of graduate students, faculty, and staff at Stony Brook University. The donor pool comprised approximately equal numbers of males and females, ranging from 18 to 55. Blood donors were used solely as a source of red blood cells. Healthy donors of any race or ethnicity or gender who were not taking medications were free to participate.
Recruitment	Donors were recruited by word of mouth. Blood donors were used solely as a source of red blood cells and any selection biases are not relevant for the experiments in this study.
Ethics oversight	Institutional Review Board of Stony Brook University

Note that full information on the approval of the study protocol must also be provided in the manuscript.
